# Psychological distress and sleep quality among Sri Lankan medical students during an economic crisis

**DOI:** 10.1371/journal.pone.0304338

**Published:** 2024-06-25

**Authors:** Guwani Liyanage, D. P. R. W. Rajapakshe, D. R. Wijayaratna, J. A. I. P. Jayakody, K. A. M. C. Gunaratne, A. M. A. D. K. Alagiyawanna

**Affiliations:** 1 Faculty of Medical Sciences, Department of Paediatrics, University of Sri Jayewardenepura, Nugegoda, Sri Lanka; 2 Faculty of Medical Sciences, University of Sri Jayewardenepura, Nugegoda, Sri Lanka; 3 Faculty of Medical Sciences, Department of Community Medicine, University of Sri Jayewardenepura, Nugegoda, Sri Lanka; University of New South Wales - Kensington Campus: University of New South Wales, AUSTRALIA

## Abstract

**Objectives:**

This study examined the evidence of the prevalence of psychological distress and poor sleep among medical students and its associations during an economic crisis.

**Design:**

This was a cross-sectional study using an online questionnaire. It included the Depression Anxiety Stress Scales and Pittsburgh Sleep Quality Index (PSQI).

**Setting and participants:**

This study included medical undergraduates from a large metropolitan university in Sri Lanka.

**Primary and secondary outcome measures:**

We assessed the prevalence of psychological distress, sleep quality, and factors associated with psychological distress. To evaluate the associations, we used logistic regression.

**Results:**

The majority (69.2%) had some form of distress (depression, anxiety or stress), while 23% had distress in all three. Anxiety was the most prevalent (50.7%). Poor sleep quality was reported in 41%. The highest contribution to global PSQI was from sleep latency, duration, and daytime dysfunction subscales. In bivariate analysis, sleep quality was directly related to depression (*t*_245.65_ = -6.75, p<0.001)., anxiety (*t*_313.45_ = -6.45, *p* <0.001), and stress (*t*_94.22_ = -5.14, *p* <0.001). In multinomial logistic regression models, sleep quality was independently associated with depression, anxiety and stress. In addition, frequent contact with friends was inversely associated with depression and anxiety. Also, social media use was inversely linked to depression, anxiety and stress. Clinical-year students had lower depression and anxiety than non-clinical students. Engaging in mindfulness activities was inversely associated with depression. However, models explained only a moderate amount of variance (Nagelkerke R-squared values were 0.21, 0.18, and 0.13 for depression, anxiety and stress, respectively).

**Conclusions:**

Poor sleep quality and psychological distress are high among the undergraduates. The findings emphasize the importance of addressing sleep quality and psychological wellbeing in medical undergraduates. Further research with larger and more diverse samples is needed for a more comprehensive understanding of the factors associated with psychological distress among undergraduates.

## Introduction

The transition from high school to university life is exciting and challenging. Although undergraduate education allows personal growth, fulfilment, and wellbeing, poor adjustment and stress unintentionally negatively affect students’ psychological wellbeing. Predicting how each student will adjust to the new environment is not always possible. In particular, medical schools undertake an extensive selection process to identify prospective candidates with a strong commitment. Their workload is heavier than undergraduates from other faculties, and they have less time for family and friends, hobbies, or personal care. Burnout is frequent and significantly related to heavy workload [1]. Thus, it is reported that medical students experience more mental health issues than their matched peers [2–4].

A high prevalence of depression, anxiety, and stress among undergraduate students is reported worldwide [2,5–8]. In addition, poor sleep quality and excessive daytime sleepiness among undergraduates have been reported [9,10]. Most importantly, sleep quality is described as having a bidirectional connection with psychological wellbeing [11]. Also, undergraduates’ psychological issues are linked to socioeconomic conditions, social interactions, academic pressure, coping mechanisms and many other biological and contextual factors [2,12,13].

Studies on psychological distress in Sri Lankan undergraduates are few, with even fewer exploring the association between sleep quality and psychological distress [2,3,8,14]. Further, in early 2022, Sri Lankans started experiencing economic and political instability in the country, the worst economic crisis of its history [15]. The education sector was already in crisis before the current economic conditions due to the Covid-19 pandemic. Although some reform areas were getting adequate attention, education received less consideration during the economic crisis. Thus, our main aim was to explore the rate of psychological distress and sleep quality among undergraduate medical students during the ongoing economic crisis. In addition, we also aimed to evaluate factors associated with psychological distress.

## Methods

### Participants

A cross-sectional descriptive study was conducted among the medical undergraduates at a large metropolitan medical school in Sri Lanka. Each year, a diverse group of students from all parts of the island are registered for the five-year MBBS programme at this university. This study was conducted from May 2022 to October 2022. Students with chronic physical illnesses (e.g., chronic kidney disease, chronic liver disease, etc.) and psychiatric illnesses were excluded. The sample size was calculated considering a confidence interval of 95%, a precision of 0.05, and an expected proportion of 64% [2]. Including a non-response rate of 20%, the required sample size was 420. This study was approved by the Ethics Committee of the Faculty of Medical Sciences, University of Sri Jayewardenepura, Sri Lanka (Reference number: CM/29/22).

### Study instruments

The questionnaire (S1 Appendix) contained four parts. Part 1: General information including socio-demographic data, Part 2: Depression Anxiety Stress Scales (DASS-21), Part 3: Pittsburgh Sleep Quality Index (PSQI), and Part 4: Individual factors likely to be associated with psychological distress.

DASS-21 is a validated tool to evaluate psychological distress in clinical and non-clinical settings [16,17]. The DASS-21 is a measure of depression, anxiety and stress. Each component is assessed with 14 items. The participants’ experience over the past week was evaluated on a 4-point severity/frequency scale. The total DASS-21 score for each item was categorized according to the DASS manual. (normal, mild, moderate, severe and extremely severe) [16,17].

The PSQI is a 19-item questionnaire that measures sleep quality and disturbance over the past month [18]. It has seven components (sleep duration, sleep disturbance, sleep latency, daytime dysfunction due to sleepiness, sleep efficiency, overall sleep quality, and sleep medication use). The score ranges from 0 to 21, with the higher total score indicating worse sleep quality. A global PSQI score >5 indicates poor quality sleep and has a sensitivity of 89.6% and a specificity of 86.5% [18].

The first and fourth sections of the questionnaire were piloted among the participants to test their clarity and verify their overall comprehensibility. Relevant sections were modified according to the comments.

### Procedure

An online questionnaire was designed using Google Forms. All registered students from 1^st^ to 5^th^ year were invited via WhatsApp/email. Reminders were sent to all students requesting to fill in the questionnaire. This Google form contained the information sheet screening questions and consent to participate. The screening questionnaire obtained information on general health and chronic illnesses. Written informed consent was obtained from the participants. All participants were informed that their participation would only be voluntary; they could withdraw at any time, and the data was anonymous. All participants were adequately informed about the study before consenting to participate. Google Forms settings were adjusted so that an individual could send only one response, avoiding a single person giving multiple responses. Information is available only to those who have responded.

### Statistical analysis

All statistical analyses were done with the Statistical Package for Social Sciences version 22. Data was checked for normality, and outliers were removed. Non-parametric tests were used to compare median differences. The Cronbach alpha coefficient for the DASS-21 score for the present study was 0.94. In a previous validation study, Cronbach alpha coefficient was 0.89 [19]. Cronbach alpha coefficient for the PSQI score for the current study was 0.64 and 0.75 in a validation study [20].

The binary logistic regression analysis was carried out to assess predictors of psychological distress. The dependent variable (DASS-21 score) was dichotomized as normal/mild and moderate/severe/extremely severe. Independent variables were age, gender, educational background of mother and father, total household income, travel from home/not, clinical years/not, and individual factors such as social media usage. We hypothesized that medical students in clinical years would report higher mean scores on the DASS-21 than non-clinical students. Mothers’/fathers’ education was classified as primary (Grades 1–5), secondary (Grades 6–13), and tertiary. We used a collapsed version for education (secondary education/less and post-secondary education) since less than ten parents had only primary education. Household income was dichotomized based on the household income data in Sri Lanka [21]. Ordinal independent variables (e.g., social media usage) were treated as continuous. Independent variables were checked for multicollinearity. The backward conditional multinomial logistic regression was performed to produce a model for each domain of DASS-21. The results were given as an adjusted odd ratio (aOR) with a 95% confidence interval.

## Results

A total of 422 medical students responded to the online survey. Table 1 shows the basic characteristics of the participants. The majority were females (68.5 %). Most parents had post-secondary education. One-fifth of total household income was less than the median income. One-third (21%) were travelling from home.

**Table 1:** Characteristics of the study population.

Characteristic	n (%)
**Age** (years) **mean±SD**	24.3 (1.97)
**Gender (Male)**	133 (31.5)
**Study year (Clinical)**	230 (54.5)
**Education (Mother)**	
Primary	08 (5.0)
Secondary	31 (19.5)
Post-secondary	120 (75.5)
**Education (Father)**	
Primary	04 (0.9)
Secondary	199 (47.2)
Post-secondary	219 (51.9)
**Household income** (LKR)	
< Median	87 (20.6)
≥Median	335 (79.4)
**Travelling from home (Yes)**	130 (30.8)

Figures are presented as n (%) unless otherwise stated.

Abbreviations: LKR: Sri Lankan Rupees, SD: Standard deviation.

### Psychological distress

The median (IQR) for DASS-21 subscales for depression, anxiety and stress were 10 (IQR: 4, 14), 8 (IQR:2,12) and 8 (IQR:4,14), respectively. The majority (69.2%) had mild/moderate/severe distress in one/or more of the domains, while 23% had distress in all three domains. Anxiety was the most prevalent type among participants (50.7%). Depression was noted in 50%, while only 42.2% suffered from stress (Table 2). Clinical students had lower mean DASS-21 scores than the non-clinical students (mean difference 8.55, p = 0.001).

**Table 2:** Severity of DASS-21 scores among the participants.

	Depression	Anxiety	Stress
Normal	211 (50)	208 (49.3)	244 (57.8)
Mild	66 (15.6)	45 (10.7)	108 (25.6)
Moderate	87 (20.6)	103 (24.4)	50 (11.8)
Severe	32 (7.6)	34 (8.1)	15 (3.6)
Very severe	26 (6.2)	32 (7.6)	5 (1.2)

### Sleep quality indicators

**Fig 1:**
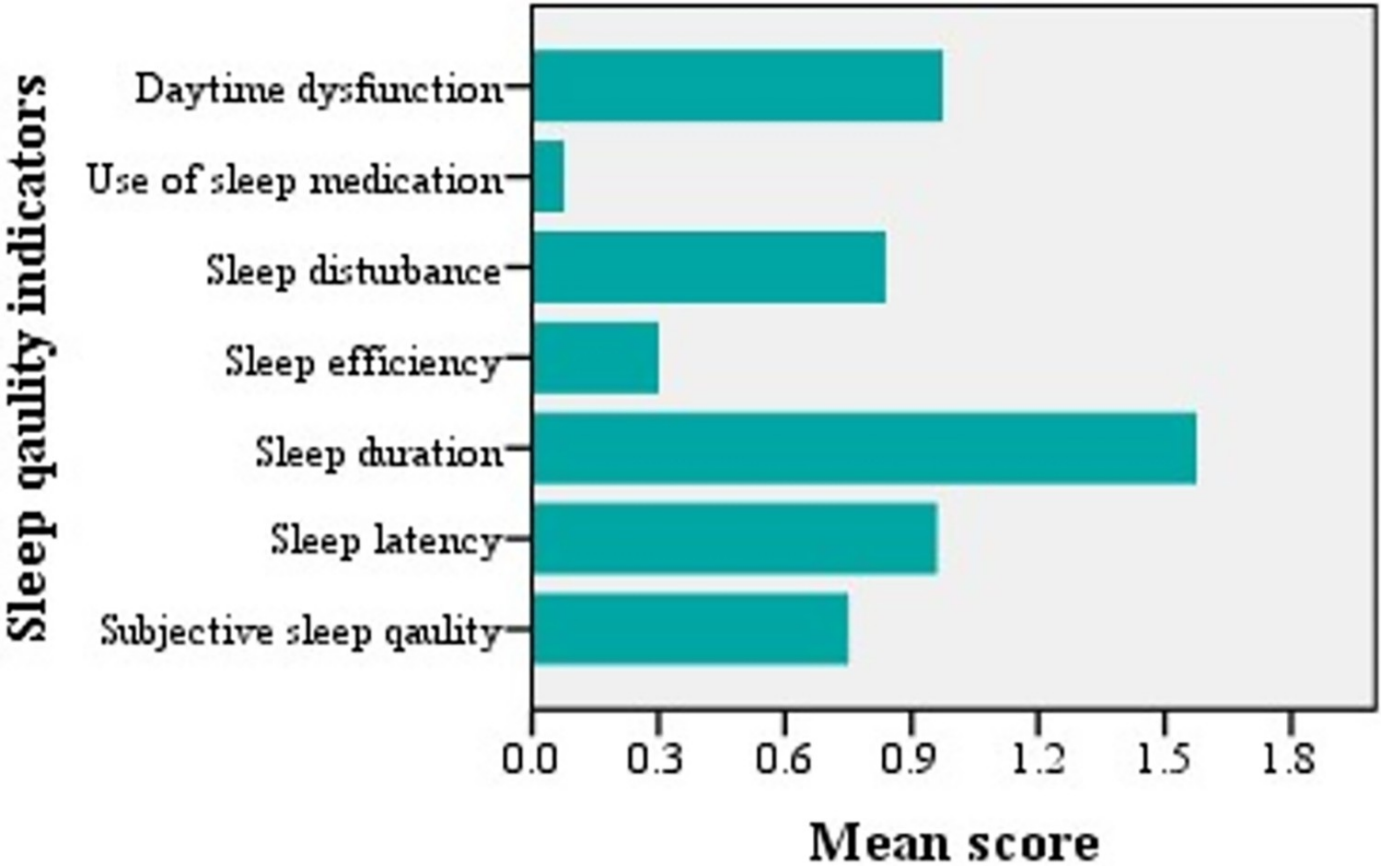
Sleep quality indicators among study subjects (higher mean scores indicating worse sleep quality).

The rate of overall poor sleep quality (PSQI score > 5) was 41% (n = 173). The mean score ≥1 was noted for sleep latency, duration, and daytime dysfunction, and they were the main items contributing to the total PSQI (Fig 1). Scores for sleep efficiency and use of sleep medications were lower than those for the other domains. The clinical students had better sleep quality than non-clinical students (mean difference: 1, p = 0.003). In bivariate analysis, sleep quality was significantly associated with depression (*t*_245.65_ = -6.75, p <0.001)., anxiety (*t*_313.45_ = -6.45, *p* <0.001), and stress (*t*_94.22_ = 5.14, *p* <0.001). S1 Appendix shows the association between each component of sleep quality and DASS-21 scores. Except for sleep duration and sleep medication, other components showed a statistically significant association with depression, anxiety and stress.

### Predictors of psychological distress

The bivariate analysis between three domains of psychological distress and predictor variables is given in Table 3, and multinomial logistic regression in Table 4. Overall sleep quality was independently associated with all three domains of psychological distress. Frequent contact with friends reduced depression and anxiety. Frequent social media use increases the risk of moderate/severe depression, anxiety, and stress. Students in clinical years had moderate/severe depression and anxiety than their non-clinical colleagues. Engaging in mindfulness activities was protective against depression.

**Table 3:** Summary of results of bivariate analysis of study variables with three Depression Anxiety Stress Scale (DASS-21) sub-scale scores.

Study variable	Depression	Anxiety	Stress
Age^a^	2.07 (0.04)	1.58 (0.1)	1.20 (0.3)
Gender^b^	2.19 (0.14)	0.03 (0.8)	0.34 (0.5)
Study year^b^	16.39 (0.001)	13.60 (<0.001)	3.77 (0.05)
Mother’s education^b^	0.96 (0.3)	1.19 (0.2)	0.32 (0.5)
Father’s education^b^	0.024 (0.8)	0.28 (0.5)	1.49 (0.2)
Household income^b^	0.43 (0.8)	0.71 (0.7)	2.18 (0.13)
Travelling from home^b^	0.022 (0.8)	2.49 (0.1)	1.70 (0.1)
PSQI score^a^	-7.08 (<0.001)	-6.63 (<0.001)	-5.19 (<0.001)
Hobbies/ sports activities^c^	18301.00 (0.08)	18998.00 (0.02)	11329.00 (0.2)
Mindfulness activities^c^	16928.00 (0.007)	18793.00 (0.03)	10775.00 (0.09)
Social media usage^c^	17608.00 (0.03)	19453.00 (0.09)	9798.50 (0.004)
Contact with friends^c^	17289.00 (0.01)	17979.50 (0.004)	12183–50 (0.9)
Contact with family^c^	17169.50 (0.008)	17204.50 (<0.001)	10643.00 (0.05)
Romantic relationships^c^ (Yes)	1.57 (0.4)	1.76 (0.4)	0.704 (0.7)

^a^ Independent t-test, One-way ANOVA

^b^ Chi-square test

^c^ Mann-Whitney U test.

Abbreviations: PSQI-Pittsburgh Sleep Quality Index.

All three domains of DASS-21 were dichotomized to moderate/severe vs normal/mild.

**Table 4:** Factors associated with depression, anxiety, and stress among participants using multinomial logistic regression.

	AOR	95% CI		P value
		Lower	Upper	
**Depression**				
PSQI score	1.28	1.279	1.174	<0.001
Gender (Male)	0.62	0.374	1.020	0.06
Study year (Clinical)	0.59	0.374	0.923	0.02
Contact with friends	0.700	0.546	0.889	0.004
Social media usage	1.23	1.027	1.628	0.03
Mindfulness activities	0.80	0.675	0.961	0.02
(χ2 = 68.29, df = 6, p-value <0.001, Nagelkerke R2 = 0.21)				
**Anxiety**				
PSQI score	1.25	1.159	1.366	<0.001
Study year (Clinical)	0.63	0.407	0.960	0.03
Contact with family	0.75	0.587	0.967	0.03
Contact with friends	0.77	0.597	0.981	0.04
Social media usage	1.32	1.052	1.644	0.03
(χ2 = 60.50, df = 5, p-value <0.001, Nagelkerke R2 = 0.18)				
**Stress**				
PSQI score	1.27	1.155	1.400	<0.001
Social media usage	1.43	1.074	1.891	0.01
Income (Less than the median)	1.74	0.922	3.237	0.08

(χ2 = 33.19, df = 3, p-value <0.001, Nagelkerke R2 = 0.13).

## Discussion

This study reports a high level of psychological distress and poor sleep quality among medical students. The distress is expected to be more pronounced during times of crisis, such as pandemics, economic crunch, natural disasters or other times of heightened stress [22]. Yet, exact comparisons between our study and published studies are arbitrary due to differences in metrics, sampling techniques, populations, etc.

We observed 69.2% of medical students experiencing some form of psychological distress (mild to severe), which is seemingly high. Thus, it suggests that medical students in our study have faced significant psychological challenges during the study period. Several factors may have been the reasons for the high levels of distress: academic pressure, lack of support systems, and socio-economic factors. Before the economic crisis, a study in six Sri Lankan medical schools reported that 62% of medical students had distress using General Health Questionnaires (GHQ-12), which was lower than the reported rate in our study [3]. In contrast, a study conducted among medical students in Sri Lanka in mid-2018 (before the pandemic) reported 82% with some distress [2]. The Kessler K10 Psychological Distress scale assessed psychological distress in that study. In line with other studies in developing countries, the medical students in our study showed higher levels of depression, anxiety and stress compared to a cohort of Australian medical students [23].

During the COVID-19 pandemic, 78.2% of female medical undergraduates were psychologically distressed, which was higher than in our cohort [24]. Also, compared to the depression rate (50%) in our study, another Sri Lankan study observed 70% of the students experiencing some form of depression ranging from mild to severe among students (management studies & commerce, science and medicine) at a university in the Northern part of the country during the COVID-19 pandemic [14]. That study used the Patient Health Questionnaire (PHQ) to assess psychological distress, while we used the DASS scale. Previous studies have documented a moderate to high correlation between the DASS-21 and the PHQ [25]. With the above examples, it can be assumed that depression among Sri Lankan undergraduates during the economic crisis is lower than during the COVID-19 pandemic. The difference could have been due to pandemic-related barriers to academic routines, disruption of clinical training, and students worrying about their performance at clinical exams and postponement of exams.

In our study, 41% experienced poor sleep quality. It has been previously reported that families of low socio-economic backgrounds are linked to greater sleep problems [26]. Also, knowledge gaps regarding good sleep hygiene, poor stress management techniques, and low access to mental health resources could be other reasons for low sleep hygiene in medical students. Variable rates of sleep quality in university undergraduates have been reported globally, as high as 60% in some countries [27]. A previous study among 800 medical students in Sri Lanka reported a lower rate of poor sleep quality (25.9%) using similar metrics in a single centre in Sri Lanka [28]. Therefore, the results from the current study are a good eye-opener, highlighting the importance of providing adequate support and resources to promote good sleep hygiene among Sri Lankan medical students.

In our study cohort, sleep quality was significantly related to mental wellbeing after adjusting for covariates. Likewise, many studies have shown that sleep quality significantly predicts depressive symptoms [29,30]. Sleep plays a significant role in mood and emotions based on the remarkable neurobiology of sleep, particularly rapid eye movement (REM) sleep [31]. Poor sleep also affects cognitive functions such as memory and decision-making [31]. This, in turn, can increase stress and anxiety. Moreover, poor sleep can affect the immune function and metabolism and cause poor physical health, adding to psychological distress [32]. In addition, a bidirectional association between self-reported sleep and mental health has been reported [33,34]. For example, insomnia is bi-directionally related to anxiety and depression [11]. Thus, it is evident that sleep is a fundamental aspect of physical and mental health, and promoting good sleep habits can positively impact students’ academic performance, cognitive function, emotional wellbeing, and overall quality of life [6]. A meta-analysis of randomized controlled trials reported that better sleep quality leads to more considerable improvements in mental health [35]. Thus, future studies could examine what strategies that improve sleep could be integrated into mental health services and vice versa.

In addition to sleep, many other socio-demographic and contextual factors were associated with psychological distress. We hypothesized that the socio-economic status (SES) proxy variable (household income) is associated with DASS-21 scores during the economic crisis. Yet, in the multinomial logistic regression analysis, household income did not significantly correlate with any of the domains of the DASS scores. Likewise, previous studies have reported similar findings [36]. In contrast, other studies have described increased psychological distress due to financial strain [37–39]. In addition, a systematic review and meta-analysis of over 50 studies have shown an inverse relationship between income and depression [40].

Frequent social media use increases psychological distress. Previous reports have shown that time spent, activity, number of accounts, frequency of checking for messages and addiction to social media are correlated with depression, anxiety and psychological distress [41]. The main concern with social media use is that it can lead to negative experiences such as cyberbullying, misinformation, privacy issues and comparisons resulting in psychological issues [42,43]. Contact with family and friends was protective against psychological distress in this cohort; the more frequent the contact, the lower the distress. As per previous reports, adequate social and family support can enhance emotional backing, a sense of belonging, resilience, coping mechanisms and stress reduction [44]. Similarly, engaging in mindfulness activities reduced the depressive symptoms in the study subjects. It has been shown that various stress reduction techniques and mindfulness activities are practical and efficient in reducing psychological distress [45]. A non-randomized controlled trial among psychology undergraduates using a brief mindfulness training demonstrated significantly lower levels of depression and significantly higher levels of subjective happiness in the experimental group than the control group [46]. Clinical-year medical students had lower depression and anxiety scores compared to pre-clinical students. A six-year longitudinal study in the United States showed that study-related distress is more common in preclinical than clinical years [47]. More relaxation in clinical years, the practical relevance of the topics, improved coping mechanisms with university support systems, and experience in dealing with the curriculum content with time could have been reasons for less distress in later years. No gender difference in psychological distress was observed in our study, similar to previous Sri Lankan studies indicating that both male and female students have a similar risk of psychological distress [28]. The regression models explained only a moderate amount of variance and are insufficient to draw definite conclusions on the association of factors such as social media use, contact with family and friends, and engagement in mindfulness activities with psychological distress. Other factors, such as interactions and sample size, could likely impact the association. Therefore, further exploration of this subject is required to draw concrete conclusions.

The use of validated scales increased the reliability of our study results. The high Cronbach’s alpha coefficient for the DASS-21 score in the present study (0.94) indicates excellent internal consistency reliability. Moreover, the consistency of the Cronbach’s alpha coefficient (0.89) reported in a previous validation study adds further strength to the reliability of the DASS-21 score used in the present study [20]. However, the study findings should be interpreted with the following limitations. In sampling, self-selection bias could have occurred (i.e., students with better or worse distress may have responded). Also, self-reported data collection may have caused recall and social desirability bias. A systematic error in survey respondents’ self-reported household income is possible. One solution is using multiple indicators measuring SES used in large-scale studies, yet often impractical given the time restraints in both the collection and the data analysis. Also, we did not assess the financial effects of the economic crisis on the participants’ families (e.g., parents losing their jobs). Further, the Cronbach’s alpha coefficient for the PSQI score in the current study is slightly below the conventional threshold of 0.70 for acceptable reliability, compared to 0.75 in previous studies [20]. Data were collected from one university during an economic crisis; therefore, generalizing the results among students from other universities and during times without crisis are limitations. Despite these limitations, this study adds to the knowledge gap on psychological distress and sleep quality among medical students, particularly during a crisis. To address these issues, local medical schools should improve support systems, such as counselling services, stress management programs, and wellness initiatives. Educating medical students on sleep health and psychological distress not only benefits the students themselves but also enhances patient care. Physicians prioritizing self-care and mental health are more likely to provide better care and empathize with their patients. It is an investment in both personal and professional wellbeing.

## Conclusions

Poor sleep quality and psychological distress are high among the undergraduates in this study during an economic crisis. The findings emphasize the importance of addressing sleep quality and psychological wellbeing in medical undergraduates. Further research with larger and more diverse samples is needed for a more comprehensive understanding of the factors associated with psychological distress among undergraduates.

## Supporting information

S1 Appendix (DOCX)
