# Supplementary material for: Psychological distress and sleep quality among Sri Lankan medical students during an economic crisis
Source: PLoS One. 2024 Jun 25;19(6):e0304338. doi: 10.1371/journal.pone.0304338 (PMC11198848; doi:10.1371/journal.pone.0304338)
Supplement: S1 Appendix — (DOCX) [file pone.0304338.s001.docx]

**S1 Appendix**

Association between components of Pittsburgh Sleep Quality Index and DASS-21 scores

| **Components** | **Depression** | | | **Anxiety** | | | **Stress** | | |
| --- | --- | --- | --- | --- | --- | --- | --- | --- | --- |
|  | Moderate/severe distress | Mild/no distress | p-value | Moderate/severe distress | Mild/no distress | p-value | Moderate/severe distress | Mild/no distress | p-value |
| Subjective sleep quality | 0.94 (0.67) | 0.65 (0.62) | **<0.001** | 0.92 (0.67) | 0.63 (0.61) | **<0.001** | 0.93 (0.64) | 0.71 (0.65) | **0.01** |
| Sleep latency | 1.34 (1.4) | 0.76 (1.01) | **<0.001** | 1.26 (1.36) | 0.75 (0.99) | **<0.001** | 1.43 (1.49) | 0.86 (1.08) | **<0.001** |
| Sleep duration | 1.60 (0.88) | 1.56 (0.85) | 0.6 | 1.56 (0.89) | 1.58 (0.83) | 0.7 | 1.51 (0.74) | 1.58 (0.88) | 0.5 |
| Sleep efficiency | 0.42 (0.81) | 0.23 (0.56) | **0.005** | 0.44 (0.84) | 0.20 (0.49) | **<0.001** | 0.43 (0.77) | 0.27 (0.64) | 0.06 |
| Sleep disturbance | 1.11 (0.66) | 0.69 (0.58) | **<0.001** | 1.11(0.64) | 0.65 (0.56) | **<0.001** | 1.20 (0.71) | 0.76 (0.59) | **<0.001** |
| Sleep medication | 0.11 (0.39) | 0.05 (0.31) | 0.09 | 0.12 (0.41) | 0.04(0.29) | **0.009** | 0.13 (0.45) | 0.06 (0.32) | 0.1 |
| Daytime dysfunction | 1.34 (0.82) | 0.78 (0.72) | **<0.001** | 1.25 (0.81) | 0.78 (0.74) | **<0.001** | 1.44 (0.86) | 0.88 (0.77) | **<0.001** |
| Independent t-test | | | | | | | | | |

**QUESTIONNAIRE**

Psychological well-being and sleep quality and its associated factors among undergraduates

Section 1 – General information including socio-demographic details

1. Age (in years to 31^st^ December 2022) ____________________________
2. Gender

- Male
- Female
- Other

1. University

- University of Rajarata
- University of Ruhuna
- University of Sri Jayewardenepura

1. Academic year (Year at which you sat for the Advanced level examination) –

- 1^st^
- 2^nd^
- 3^rd^
- 4^th^
- 5^th^

1. From where do you travel to the university?

- Home
- Hostel
- Boarding place
- Relative’s house

1. Total monthly income of the family (SL Rs.)

- <49,999
- 50,000 – 99,999
- 100,000 – 199,999
- 200,000- 299,000
- >300,000

1. Father’s education

- Grade 5 or less
- Ordinary level or less
- Advanced level or less
- Degree/ diploma/ postgraduate

1. Mother’s education

- Grade 5 or less
- Ordinary level or less
- Advanced level or less
- Degree/ diploma/ postgraduate

1. What sector your hometown belongs to (urban, rural, estate)

- Urban
- Rural
- Estate

Section 2 – Psychological well-being

Please read each statement and tick how much the statement applied to you over the past week. There are no right or wrong answers. Do not spend too much time on any statement.

|  | Did not apply to me at all | Applied to me to some degree, or some of the time | Applied to me to a considerable degree or a good part of time | Applied to me very much or most of the time |
| --- | --- | --- | --- | --- |
| I found it hard to wind down |  |  |  |  |
| I was aware of dryness of my mouth |  |  |  |  |
| I couldn’t seem to experience any positive feeling at all |  |  |  |  |
| I experienced breathing difficulty (e.g., excessively rapid breathing, breathlessness in the absence of physical exertion) |  |  |  |  |
| I found it difficult to work up the initiative to do things |  |  |  |  |
| I tended to over-react to situations |  |  |  |  |
| I experienced trembling (e.g., in the hands) |  |  |  |  |
| I felt that I was using a lot of nervous energy |  |  |  |  |
| I was worried about situations in which I might panic and make a fool of myself |  |  |  |  |
| I felt that I had nothing to look forward to |  |  |  |  |
| I found myself getting agitated |  |  |  |  |
| I found it difficult to relax |  |  |  |  |
| I felt downhearted and blue |  |  |  |  |
| I was intolerant of anything that kept me from getting on with what I was doing |  |  |  |  |
| I felt I was close to panic |  |  |  |  |
| I was unable to become enthusiastic about anything |  |  |  |  |
| I felt I wasn’t worth much as a person |  |  |  |  |
| I felt that I was rather touchy |  |  |  |  |
| I was aware of the action of my heart in the absence of physical exertion (e.g., sense of heart rate increase, heart missing a beat) |  |  |  |  |
| I felt scared without any good reason |  |  |  |  |
| I felt that life was meaningless |  |  |  |  |

Section 3 – Sleep quality (Pittsburgh Sleep Quality Index - PSQI)

Instructions: The following questions relate to your usual sleep habits during the past month only. Your answers should indicate the most accurate reply for the majority of days and nights in the past month. Please answer all questions.

During the past month, what time have you usually gone to bed at night? ________________

During the past month, how long (in minutes) has it usually taken for you to fall asleep each night? __________

During the past month, what time have you usually got up in the morning? _______________

During the past month, how many hours of actual sleep did you get at night? (This may be different than the number of hours you spent in bed.) ___________________

| During the past month, how often have you had trouble sleeping because you… | Not during the past month | Less than once a week | Once or twice a week | Three or more times a week |
| --- | --- | --- | --- | --- |
| Cannot get to sleep within 30 minutes |  |  |  |  |
| Wake up in the middle of the night or early morning |  |  |  |  |
| Have to get up to use the bathroom |  |  |  |  |
| Cannot breathe comfortably |  |  |  |  |
| Cough or snore loudly |  |  |  |  |
| Feel too cold |  |  |  |  |
| Feel too hot |  |  |  |  |
| Have bad dreams |  |  |  |  |
| Have pain |  |  |  |  |
| Other reason(s), please describe  _________________________________  _________________________________  how often you have had trouble sleeping because of this |  |  |  |  |
|  | Very good | Fairly good | Fairly bad | Very bad |
| During the past month, how would you rate your sleep quality overall? |  |  |  |  |
|  | Not during the past month | Less than once a week | Once or twice a week | Three or more times a week |
| During the past month, how often have you taken medicine (prescribed or “over the counter”) to help you sleep? |  |  |  |  |
| During the past month, how often have you had trouble staying awake while driving, eating meals, or engaging in social activity? |  |  |  |  |
|  | No problem at all | Only a very slight problem | Somewhat of a problem | A very big problem |
| During the past month, how much of a problem has it been for you to keep up enthusiasm to get things done? |  |  |  |  |

Section 4- Individual factors associated with psychological well-being

1. How often do you engage in any hobbies or any other activities to relieve stress? (Eg: watching movies, listening to music, singing, playing, dancing, content creation, etc.)

- Never
- Very Rarely
- Rarely
- Occasionally
- Frequently
- Very Frequently

1. Do you engage in any mindfulness activities? (Eg: meditation, yoga, praying, etc.)

- Never
- Very Rarely
- Rarely
- Occasionally
- Frequently
- Very Frequently

1. How often do you engage in social media for non-academic purposes?

- Never
- Very Rarely
- Rarely
- Occasionally
- Frequently
- Very Frequently

1. How often do you have contact with your friends (in person or virtual)?

- Never
- Very Rarely
- Rarely
- Occasionally
- Frequently
- Very Frequently

1. How often do you have contact with your family (in person or virtual)?

- Never
- Very Rarely
- Rarely
- Occasionally
- Frequently
- Very Frequently

1. Are you currently involved in a romantic relationship?

- Yes
- No
- Rather not say
